# Non-Invasive Bioluminescence Imaging to Monitor the Immunological Control of a Plasmablastic Lymphoma-Like B Cell Neoplasia after Hematopoietic Cell Transplantation

**DOI:** 10.1371/journal.pone.0081320

**Published:** 2013-12-12

**Authors:** Martin Chopra, Sabrina Kraus, Stefanie Schwinn, Miriam Ritz, Katharina Mattenheimer, Anja Mottok, Andreas Rosenwald, Hermann Einsele, Andreas Beilhack

**Affiliations:** 1 Department of Internal Medicine II, University Hospital Würzburg, Würzburg, Germany; 2 Center for Interdisciplinary Clinical Research, Würzburg University, Würzburg, Germany; 3 Institute of Pathology, Würzburg University, Würzburg, Germany; Columbia University, United States of America

## Abstract

To promote cancer research and to develop innovative therapies, refined pre-clinical mouse tumor models that mimic the actual disease in humans are of dire need. A number of neoplasms along the B cell lineage are commonly initiated by a translocation recombining c-myc with the immunoglobulin heavy-chain gene locus. The translocation is modeled in the C.129S1-Igha^tm1(Myc)Janz^/J mouse which has been previously engineered to express c-myc under the control of the endogenous IgH promoter. This transgenic mouse exhibits B cell hyperplasia and develops diverse B cell tumors. We have isolated tumor cells from the spleen of a C.129S1-Igha^tm1(Myc)Janz^/J mouse that spontaneously developed a plasmablastic lymphoma-like disease. These cells were cultured, transduced to express eGFP and firefly luciferase, and gave rise to a highly aggressive, transplantable B cell lymphoma cell line, termed IM380. This model bears several advantages over other models as it is genetically induced and mimics the translocation that is detectable in a number of human B cell lymphomas. The growth of the tumor cells, their dissemination, and response to treatment within immunocompetent hosts can be imaged non-invasively *in vivo* due to their expression of firefly luciferase. IM380 cells are radioresistant *in vivo* and mice with established tumors can be allogeneically transplanted to analyze graft-versus-tumor effects of transplanted T cells. Allogeneic hematopoietic stem cell transplantation of tumor-bearing mice results in prolonged survival. These traits make the IM380 model very valuable for the study of B cell lymphoma pathophysiology and for the development of innovative cancer therapies.

## Introduction

To assess the efficacy of novel anti-cancer therapies, refined pre-clinical mouse models that mimic the actual disease in humans need to be developed [1]. Translocations recombining C-MYC at 8q24 with the immunoglobulin heavy-chain gene locus, IGH, at 14q32 (8;14)(q24;q32) in B cells are seen as the initiating genomic event in Burkitt's lymphoma and this translocation is also observed in other aggressive B cell tumors such as acute lymphoblastic leukemia [6,7] and plasmacytoma/multiple myeloma [8,9]. The (8;14)(q24;q32) translocation is modeled in the C.129S1-*Igha* ^tm1(Myc)Janz^/J mouse [10]. Tumor-free heterozygous mice exhibit increased B cell proliferation and apoptosis and have enlarged lymph nodes and spleen due to follicular hyperplasia. More than two thirds of these mice develop mature B cell tumors between the age of 6 and 21 months resembling human endemic Burkitt's lymphoma or plasmacytoma [10].

To reliably evaluate the response of deep-tissue tumors dynamically, non-invasive imaging techniques have gained widespread acceptance, and among these, bioluminescence imaging has proven a valuable tool to assess tumor growth *in vivo* [11–14].

In the current study, we have isolated tumor cells from the spleen of a C.129S1-*Igha* ^tm1(Myc)Janz^/J mouse that spontaneously developed a plasmablastic lymphoma-like neoplasia. The cells were transduced to stably express eGFP and firefly luciferase and gave rise to a highly aggressive, transplantable B cell lymphoma cell line, termed IM380. Exploiting *in vivo* bioluminescence imaging, we could assess homing of lymphoma cells to lympho-hematopoietic compartments and their response to allogeneic stem cell transplantation.

## Materials and Methods

### Ethics statement

All experiments were performed according to the German regulations for animal experimentation. The study was approved by the Regierung von Unterfranken as the responsible authority (Permit Number 55.2-2531.01-103/11). All procedures were performed under esketamine/xylazine anesthesia, and all efforts were made to minimize suffering.

### Animals

BALB/c and C57Bl/6 mice were obtained from Charles River (Sulzfeld, Germany). C.129S1-Igha^tm1(myc)Janz^/J (in BALB/c H-2^d^ background) mice expressing mouse c-myc under the control of the endogenous immunoglobulin heavy-chain 2 C-alpha locus [10] were initially obtained from Jackson Laboratories (Bar Harbor, ME, USA). Female BALB/c and C57Bl/6 mice were used for experiments between 8 and 12 weeks of age. All mice were kept within the specified pathogen-free animal facility of the Center for Experimental Molecular Medicine at the Würzburg University Hospital receiving rodent chow and autoclaved drinking water ad libitum.

### Isolation of bone marrow cells and splenic T cells from C57Bl/6 mice

Bone marrow cells were isolated by flushing femur and tibia bones with phosphate buffered saline (PBS). The cell suspension was filtered through a 70 µm cell strainer (BD, Heidelberg, Germany). Spleens were directly filtered through a 70 µm cell strainer into erythrocyte lysis buffer (168 mM NH_4_Cl, 10 mM KHCO_3_, 0.1 mM ethylenediaminetetraacetic acid (EDTA)), incubated for 2 minutes, and 2 volumes of PBS were added to the single cell suspension. The cells were spun down; the cell pellet was resuspended in PBS and filtered through a new 70 µm cell strainer, before being spun down again. The resulting pellet was resuspended in PBS and cells were used for further experiments. T cells were enriched from splenocytes using the Dynal Mouse T cell Negative Isolation Kit (Invitrogen, Darmstadt, Germany) according to the manufacturer's instructions.

### Generation of a luciferase-expressing malignant plasmablastic lymphoma-like B cell line

C.129S1-Igha^tm1(myc)Janz^/J mice exhibit B cell hyperproliferation and develop B cell and plasma cell neoplasms, the incidence of which increases with age [10]. Splenocytes from a five months old female moribund mouse (internal number 380) were isolated and cultured in RPMI medium supplemented with 10% fetal bovine serum (FBS), 1% antibiotics (penicillin, streptomycin), L-glutamine and 0.1% β-mercaptoethanol (cRPMI) for six weeks and passaged once weekly to allow malignant clones to emerge. For lentiviral transduction, 293 T cells were transiently transfected with a standard calcium phosphate precipitation protocol in 10 cm dishes using 10 µg pMDL and 5 µg RSV-REV packaging plasmids, 6 µg VSV/G envelope plasmid and 20 µg of the target plasmid FUGLW (FUGW plasmid (Addgene, Cambridge, MA) with the firefly luciferase inserted into it). Two days later, the supernatant containing the lentiviral particles was harvested, filtered through a 0.45 µm filter, 8 µg polybrene/ml were added and the mixture was used to transduce the IgH-myc tumor cells. The transduced cells were flow sorted twice for eGFP-expression and were termed IM380 (IgH-myc-induced lymphoma 380). For *in vivo* experiments, 10^5^ IM380 cells in 100 µl PBS were injected into the lateral tail vein of female syngeneic BALB/c mice.

### Immunoglobulin typing

The immunoglobulin isotype of IM380 cells was determined using the IsoQuick Kit for Mouse Monoclonal Antibodies (Sigma, Schnelldorf, Germany) according to the manufacturer's instructions.

### Cytotoxicity assay

IM380 cells were cultured in 96 well plates for 48 h with different concentrations of various cytotoxic drugs before staining with annexin V-Pacific Blue (Biolegend, Fell, Germany) and propidium iodide and flow cytometry. Survival rates for treated cultures were normalized to the survival rate of untreated cultures to generate survival inhibition curves and to calculate IC_50_-values for the individual drugs [14]. To assess for cytotoxic killing of tumor cells (H-2^d^) by allogeneic C57Bl/6 (H-2^b^) T cells, T cells were isolated and activated with αCD28 (2 µg/ml) and plate-bound αCD3 for 48 h. IM380 cells (5000 per well) were co-cultured with activated T cells at indicated ratios for 72 h in a round bottom 96-well plate. Tumor cell numbers were assessed by their *in vitro* bioluminescence.

### Allogeneic stem cell transplantation and graft-versus-tumor model

Tumor-bearing BALB/c mice were lethally irradiated using a Faxitron CP-160 X-ray irradiation system (Faxitron X-Ray, Lincolnshire, IL, USA) with a dose of 8 Gy [15] six days after tumor cell inoculation. Irradiated mice were injected with 5×10^6^ allogeneic bone marrow cells and 5×10^5^ enriched T cells isolated from C57Bl/6 donor mice intravenously into the retro-orbital plexus. Mice were treated with antibiotic drinking water (Baytril, Bayer, Leverkusen, Germany) for one week to prevent infections following lethal irradiation.

### *In vivo* and *ex vivo* bioluminescence imaging

For *in vivo* bioluminescence imaging [15,16], mice were anesthetized with an intraperitoneal injection of 80 mg/kg body weight (bw) esketamine hydrochloride (Pfizer, Berlin, Germany) and 16 mg/kg bw xylazine (cpPharma, Burgdorf, Germany). Together with anaesthetics, mice were injected with 300 mg/kg bw D-luciferin (Biosynth, Staad, Switzerland). Ten minutes later, bioluminescence signals of the anesthetized mice were recorded using an IVIS Spectrum imaging system (Perkin-Elmer/Caliper Life Sciences, Mainz, Germany). Pictures were taken from the ventral view in automatic mode with a maximum exposure time of five minutes per picture. For *ex vivo* imaging, mice were injected with D-luciferin and euthanized 10 minutes later. Internal organs were removed and subjected to *ex vivo* bioluminescence imaging. Pictures were evaluated using Living Image 4.0 software (Caliper Life Sciences). Tissue samples were fixed in 4% PFA, bones were subsequently decalcified for 72 h in EDTA (Titriplex, VWR, Ismaning, Germany) and embedded in paraffin for further histopathological evaluation. 2 µm sections were stained with haematoxylin/eosin and representative pictures were taken using a Nikon Eclipse E600 microscope and a Nikon DS-Fi1 camera.

### Flow cytometry

Cells were blocked with normal rat serum (1∶20 in PBS) and stained with appropriate antibodies at 4°C for 30 min. For intracellular stainings, cells were fixed and permeabilized using Fixation/Permeabilization Concentrate and Diluent from eBioscience (Frankfurt, Germany). Antibodies were obtained from either eBioscience, Biolegend, Bioss (Woburn, MA, USA), or BD (Heidelberg, Germany): B220-Biotin (RA3-6B2), BCL6-PE (mGI191E), CD10-PE/Cy7 (bs-0527R), CD138-Biotin (281-2), CD19-APC (6D5), CD20-APC (2H7), CD27-PE (LG.7F9), CD29-PE (HMβ1-1), CD38-Alexa647 (90), CD44-PE (IM7), CD49d-Alexa647 (RI-2), CD54-PE (YN1/1.7.4), H-2k^d^-Biotin (SF1-1.1), I-A^d^-Biotin (39-10-8). Biotinylated antibodies were detected with Streptavidin-Alexa647 (Invitrogen). All experiments were analyzed on a BD FACS Canto II (BD) and sample data was recorded using BD FACS Diva software and analyzed using FlowJo software (Tree Star, Ashland, OR, USA).

### Statistics

The number of animals is indicated in the figure legends. All data are shown as mean ± standard error of mean (S.E.M.). Figures were prepared with GraphPad Prism 5 software (La Jolla, CA, USA) and Adobe Photoshop 7 (San Jose, CA, USA). All groups were compared to the respective control group by two-tailed unpaired student's t-test with GraphPad InStat 3 software. Data reaching statistical significance are indicated as: * p≥0.05, ** p≥0.01.

## Results and Discussion

**Figure 1:**
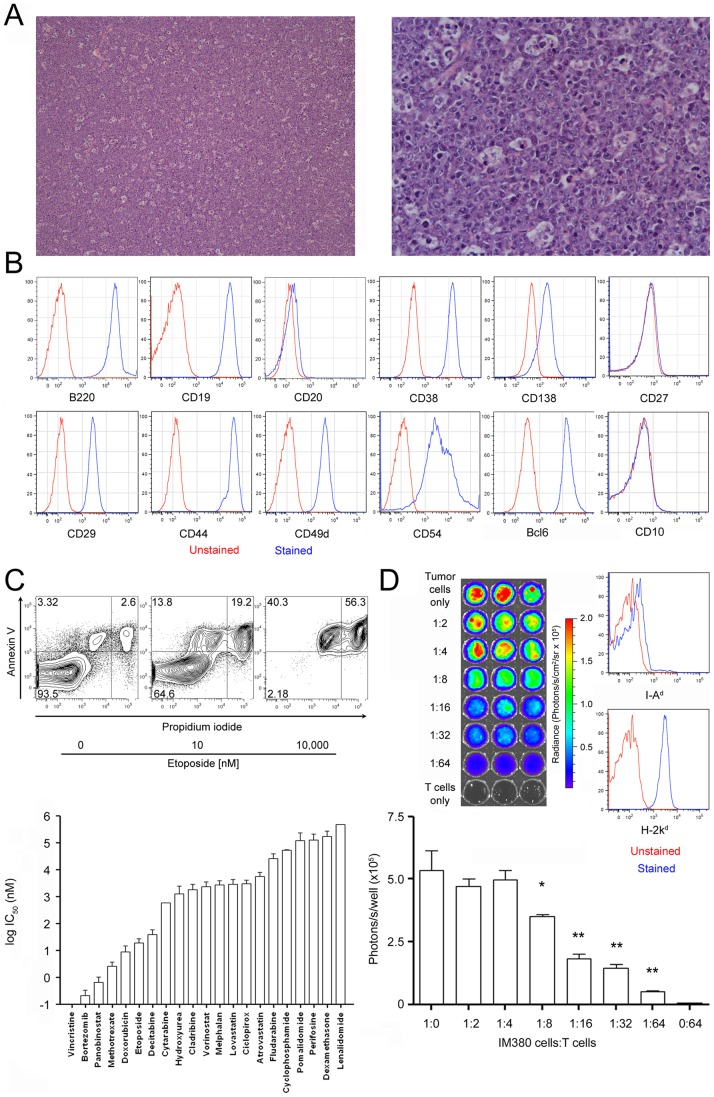
Generation of the malignantIgH-myc-driven plasmablastic lymphoma-like B cell line IM380. **A**: Photomicrograph of the initial tumor in a five months old C.129S1-*Igha* ^tm1(Myc)Janz^/J mouse. The abdominal tumor mass shows starry sky-like areas indicative of widespread apoptosis and infiltrating macrophages. The left picture shows 100× and the right picture 400× magnification, H and E staining. **B**: Malignant splenocytes were cultured *in vitro* and gave rise to the IM380 cell line that was characterized for its expression of various B cell markers and activation-associated proteins by flow cytometry. (Representative results from at least two independent experiments). **C**: IM380 cells were treated *in vitro* with different chemotherapeutics for 48 h before being subjected to annexin V/propidium iodide staining. Upper panel: Exemplary flow cytometry data for etoposide treatment. Lower panel: The graph shows the sensitivity of the cells towards the different compounds, expressed as their respective IC_50_-values. (Mean ± SEM; combined data from four independent experiments). **D**: Luciferase-transgenic IM380 tumor cells were co-cultured with activated T cells for 72 h. Tumor cell numbers were assessed by their *in vitro* bioluminescence (upper panel and graphic evaluation in lower panel). Co-cultures were set up in triplicates each and compared to the 1∶1 culture. Flow cytometric assessment of MHC expression on IM380 cells. (Representative results from two independent experiments).

We isolated splenocytes from an enlarged spleen of a moribund 5 months old heterozygous C.129S1-*Igha* ^tm1(Myc)Janz^/J mouse and subcultured these cells for six weeks. The primary tumor in the spleen and as an abdominal tumor mass showed a starry sky-like appearance indicative of widespread apoptosis and infiltrating macrophages often associated with Burkitt's lymphoma [17] but also not uncommon in plasmablastic lymphomas and advanced-stage plasmacytomas 18,19 (Figure 1A). The cultured IM380 cells doubled each 20.1±6.1 hours, they produced IgM antibodies with the κ light chain (data not shown). To characterize the immunophenotype of IM380 cells, we assessed the expression of a number of B cell markers and activation-associated surface proteins (Figure 1B). The cells expressed the pan-B cell markers B220 and CD19, but were only slightly positive for the plasma cell marker CD138. They expressed BCL6 but were negative for CD10, two markers commonly expressed by Burkitt's lymphoma [20]. IM380 cells stained negative for the B cell marker CD20, negative for CD27 which is expressed on plasma cells, and positive for the activation markers CD29, CD38, CD44, CD49d, and CD54. Loss of CD20 expression is rare in B cell lymphomas and associated with higher aggressiveness [21,22]. Burkitt's lymphomas usually do not express CD138 [23–25] or CD44 [26,27]. Lymphoblastic lymphoma usually co-expresses CD10 and CD19 but show variable expression of CD20 [28,29]. In summary, the immunophenotype of IM380 cells indicates them to be of an origin intermediate of mature B cells and plasma cells.

We next tested the responsiveness of IM380 cells towards a panel of chemotherapeutic drugs *in vitro* by the use of Annexin V/propidium iodide staining (Figure 1C). The cells displayed a spontaneous cell death rate of about 10–25% and were found to be especially sensitive towards treatment with vincristine, bortezomib, panobinostat, etoposide, decitabine, and doxorubicin. The cells were positive for H-2k^d^ (MHC class I) but negative for I-A^d^ (MHC class II). Pre-activated, but not naïve allogeneic C57Bl/6 T cells effectively killed IM380 cells *in vitro* (Figure 1D and data not shown).

**Figure 2:**
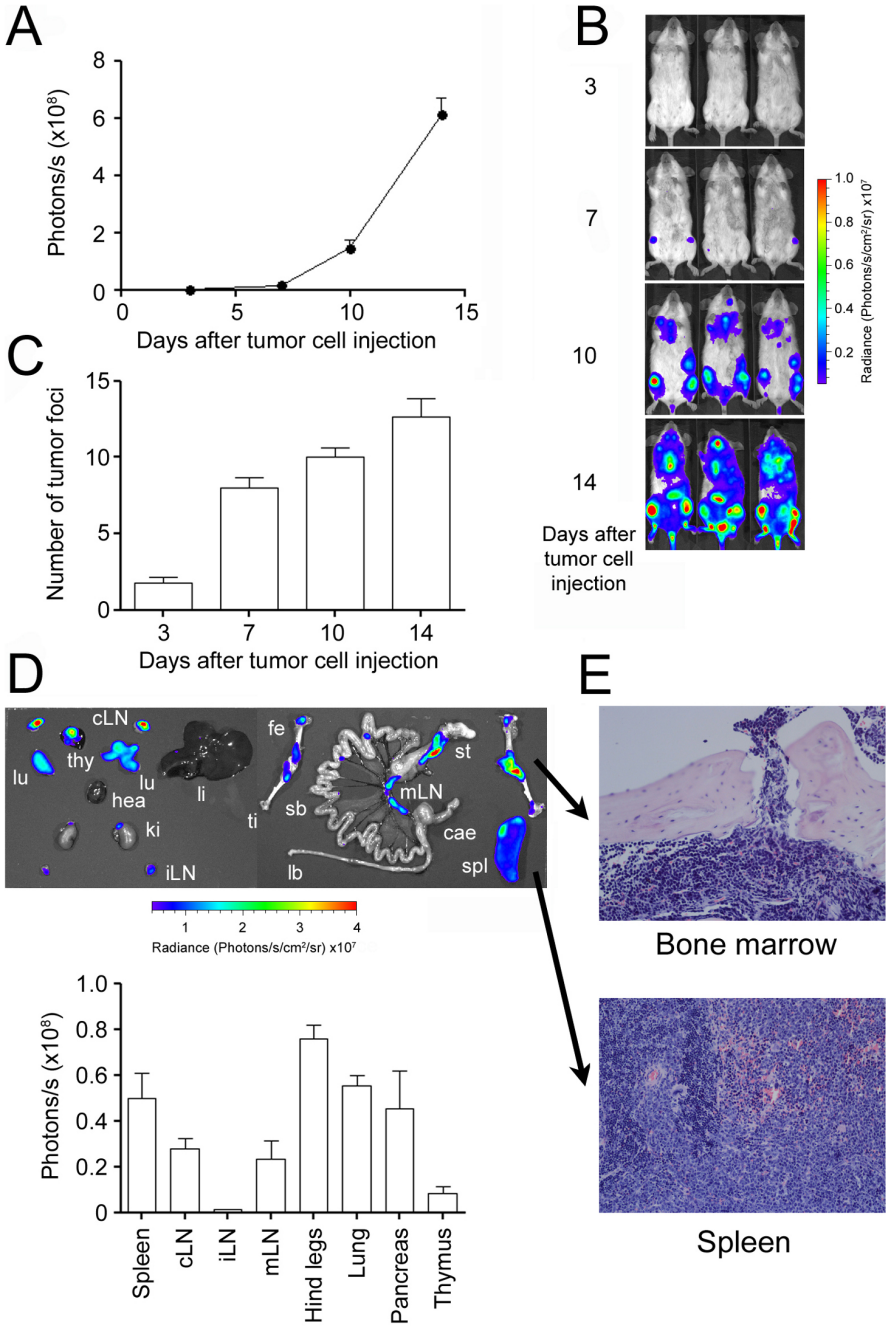
Non-invasive assessment of *in vivo* tumor growth and dissemination. **A**: 10^5^ luciferase-transgenic IM380 tumor cells were injected i.v. into the lateral tail vein into syngeneic BALB/c mice. Tumor growth was assessed by non-invasive *in vivo* BLI at the indicated time points. **B**: Representative BLI pictures of tumor-bearing mice. **C**: Tumor dissemination was determined by counting individual light-emitting tumor foci. **D**: Upper panel: Representative *ex vivo* BLI picture of a tumor bearing mouse (lu: lung, cLN: cervical lymph nodes, thy: thymus, hea: heart, ki: kidney, iLN: inguinal lymph nodes, li: liver, fe: femur, ti: tibia, sb: small bowel, lb: large bowel, mLN: mesenteric lymph nodes, st: stomach, cae: caecum, spl: spleen). Lower panel: Evaluation of tumor cell infiltration in individual organs. A–D: (Mean ± SEM; n = 5; shown is one representative experiment out of two). **E**: Representative eosin and hematoxylinstainings of organs from tumor bearing mice shown in 200× magnification.

To determine the *in vivo* homing and growth characteristics of the new IM380 cell line, we transduced the cells to stably express eGFP and firefly luciferase. Subsequently, we injected syngeneic, immunocompetent BALB/c mice with 10^5^ IM380 cells i.v. and monitored lymphoma homing and progression by non-invasive bioluminescence imaging (Figure 2A and B). IM380 lymphoma cells grew in the bone marrow compartment and within secondary lymphatic organs (spleen and lymph nodes). Tumor cell signals could be detected *in vivo* as early as two days after injection (data not shown). Over the course of the experiment, additional tumor foci could be detected which speaks for tumor dissemination of the IM380 cells as they spread from their initial sites of growth (Figure 2C). *Ex vivo* bioluminescence imaging of internal organs and hind legs (Figure 2D) showed strong tumor cell infiltration of secondary lymphatic organs (spleen>cLN>mLN>iLN), bone marrow, lungs, thymus, and pancreas. Involvement of spleen and bone marrow could also be confirmed by histology (Figure 2E).

**Figure 3:**
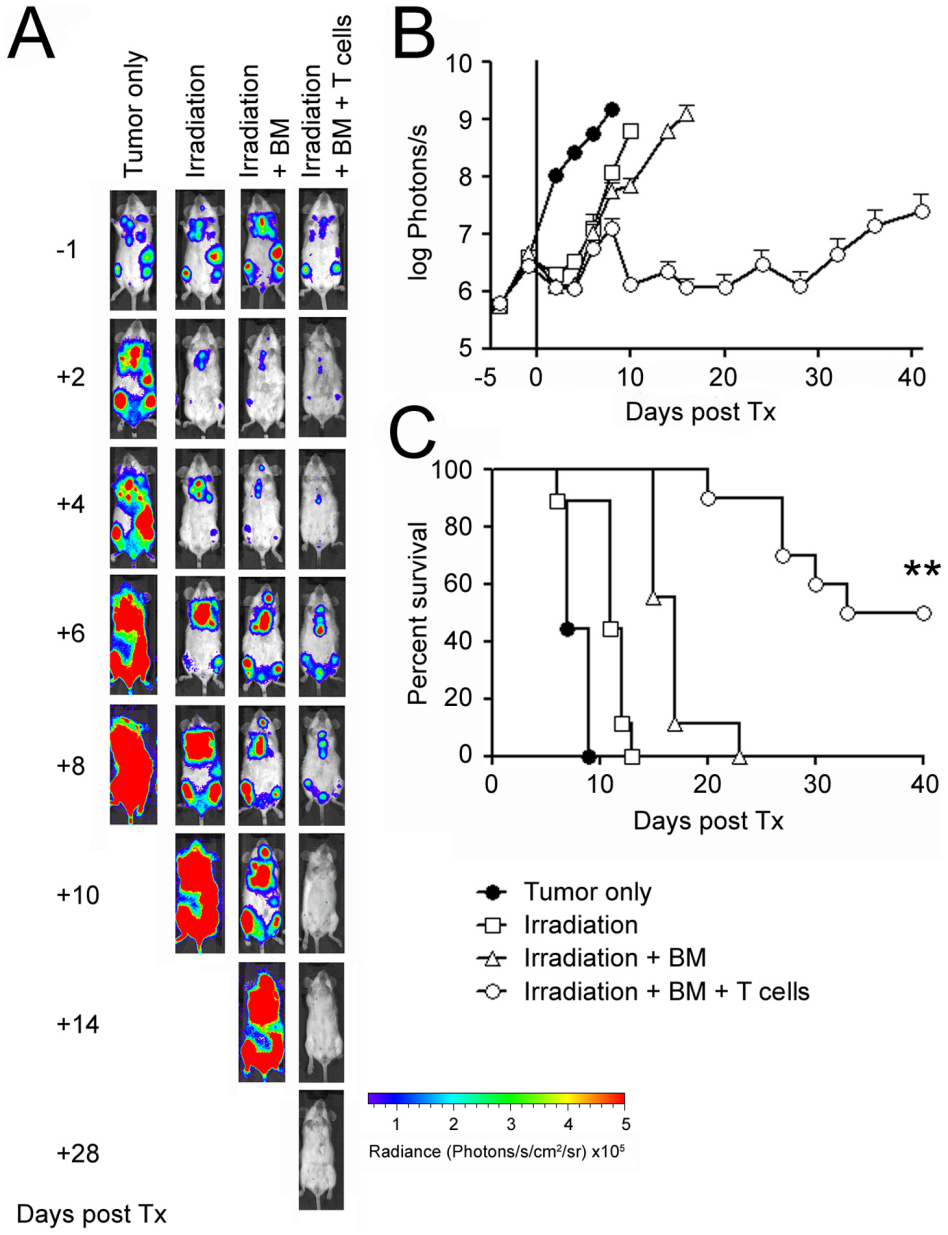
Allogeneic hematopoietic stem cell transplantation of tumor-bearing mice results in prolonged survival. 10^5^ luciferase-transgenic IM380 tumor cells were injected i.v. via the lateral tail vein into syngeneic BALB/c mice. Six days after tumor cell inoculation, mice were lethally irradiated with 8 Gy and transplanted with 5×10^6^ bone marrow cells and 0.5×10^6^ enriched splenic T cells from C57Bl/6 mice. **A and B**: Tumor growth was assessed by non-invasive *in vivo* BLI at the indicated time points (Mean ± SEM; n = 5; shown is one representative experiment out of two). **C**: Survival after allogeneic transplantation (n = 9–10; combined data from two independent experiments).

To address whether the IM380 cell line is suitable for graft-versus-tumor studies, we transplanted BALB/c mice with pre-existing tumors with allogeneic C57Bl/6 bone marrow and enriched T cells. Lethal irradiation as a pre-transplantation conditioning regimen of tumor-bearing mice resulted in tumor regression that lasted only for about four days after which the tumor relapsed and progressed. The same held true for mice that were irradiated and transplanted with allogeneic bone marrow cells. Transplantation with allogeneic T cells on the other hand resulted in efficient tumor eradication (Figure 3A and B) and prolonged survival when compared to the other groups (Figure 3C) (median survival: irradiation+bone marrow: 17 days, irradiation+bone marrow+T cells: 36.5 days; p<0.0001 as assessed by Log-rank (Mantel-Cox) test). Half of the mice (5/10) that were transplanted with allogeneic T cells nevertheless eventually succumbed to the tumor within 40 days after allogeneic transplantation owing to its high aggressiveness. Whereas activated allogeneic T cells are capable of killing IM380 cells *in vitro*, the tumor cells appear to develop immune escape variants *in vivo* resulting in tumor relapse [30,31].

The IgH-myc translocation has been mimicked in mouse models before. The challenge there is that the arising tumors are very heterogenous and furthermore, the time to onset of disease varies enormously both within and between the different mouse models [10,32]. Whereas xenogenic tumor models bear relevance in terms of responsiveness of human cancer to therapy, they completely ignore the contribution of a functional immune system to tumor control on the one hand, and tumor-induced immune suppression on the other. These facts very much limit the applicability of such models in preclinical drug testing. Inoculated IM380 tumors behave in a highly reproducible manner and they arise in immunocompetent host mice which makes the IM380 cell line very valuable in the development of efficient anti-tumoral therapies. A number of drugs are currently successfully used for the treatment of aggressive B cell lymphomas [33,34]. *In vitro* drug testing revealed several of these drugs also to be functional in killing IM380 cells (e.g. vincristine, methotrexate, doxorubicine, and etoposide). We have furthermore identified a number of drugs that are promising as cytotoxic agents for these cells (e.g. bortezomib and panobinostat).

Other B cell lymphoma cell lines, be they of human or murine origin, have arisen either spontaneously or are the result of unspecific genotoxic insults [35]. The novel IM380 cell line results from a single genetic event, namely the IgH-myc translocation and thereby allows for the study of secondary mutations and clonal evolution [36].

In summary, this new murine tumor cell model has a number of advantages over other models [35]: 1. It is genetically induced and mimics the translocation that is responsible for the majority of human Burkitt's lymphoma as well as other B cell neoplasias. 2. The tumors arising from IM380 inoculation *in vivo* are highly homogenous and reproducible. 3. Growth, dissemination, and response to treatment can be imaged non-invasively *in vivo* due to the expression of firefly luciferase by the tumor cells. 4. Established tumors can be allogeneically transplanted to study graft-versus-tumor effects of transplanted T cells as well as tumor cell immune escape. Nevertheless, the IM380 cell line is highly aggressive *in vivo* as mice succumb to it within less than three weeks following i.v. inoculation, established tumors are radiation-resistant and allogeneic transplantation is not sufficient to eradicate the tumors in all mice. These traits make the IM380 model highly valuable for the study of B cell lymphoma pathophysiology, disease progression in immunocompetent hosts and their response to treatment, and particularly for the development of innovative cancer immunotherapies.
